# Acute effects of multi-ingredient pre-workout dietary supplement on anaerobic performance in untrained men: a randomized, crossover, single blind study

**DOI:** 10.1186/s13102-024-00918-1

**Published:** 2024-06-09

**Authors:** Aleksander Drwal, Tomasz Pałka, Lukasz Tota, Szczepan Wiecha, Pavol Čech, Marek Strzała, Marcin Maciejczyk

**Affiliations:** 1 Department of Physiology and Biochemistry, University of Physical Education, Kraków, Poland; 2 Department of Physical Education and Health, Faculty in Biala Podlaska, Józef Piłsudski University of Physical Education, Warsaw, Poland; 3 Department of Educology and Sport, University of Presov, Presov, Slovakia; 4 Department of Water Sports, University of Physical Education, Kraków, Poland

**Keywords:** Wingate performance, Muscle power, Anaerobic, Dietary supplements, Caffeine

## Abstract

**Background:**

Multi-ingredient pre-workout dietary supplements (MIPS), which are combinations of different ingredients acting on different physiological mechanisms, can have a synergistic effect and improve performance. The aim of the study was to determine the acute effects of a multi-ingredient pre-workout supplement containing: beta-alanine, taurine, caffeine, L-tyrosine, and cayenne pepper (capsaicin) on anaerobic performance.

**Methods:**

A randomized, crossover, single-blind study was designed. Twelve young, healthy, untrained men aged 22.4 ± 1.44 years participated in the study. The participants performed a supramaximal all-out test (20 s Wingate test) twice, day by day, in random order: test after placebo or MIPS consumption. In both trials, the following variables were measured in the exercise test: total work performed, peak power, mean power, time to reach peak power, and power decrease.

**Results:**

MIPS was found to be effective in improving peak power (*p* = 0.009, ES = 0.77) and mean power (*p* = 0.04, ES = 0.62) in the Wingate test. However, the supplement consumption did not affect the amount of total work done (*p* = 0.10, ES = 0.48) in the test or power decrease (*p* = 0.07, ES = 0.53). The data indicate, that the improvement in anaerobic power was due to a significant improvement in pedaling speed, which was manifested in a significant improvement (i.e. shortening) in time to peak power (*p* = 0.003, ES = 0.88).

**Conclusion:**

A multi-ingredient pre-workout dietary supplement was found to be effective in improving Wingate (anaerobic) performance.

**Trial registration:**

NCT06363669, retrospectively registered on 11.04.2024 (ClinicalTrials.gov).

## Introduction

In modern competitive and recreational sport, in addition to the physical training performed, it is necessary to take into account and apply in training many other factors aimed at further improving physical performance. These include proper psychological support [1] or proper nutrition of the athlete [2]. In recent years, there has been a growing interest among physically active people in dietary supplements [3–5], and their sales have steadily increased [6]. The assumption is that dietary supplements aim to improve performance and augment training adaptations. Both supplementation with a single active substance, such as caffeine, as well as blends (in various proportions) of different active substances are used [6]. This type of dietary supplement, which is a combination of different agents, is referred to as multi-ingredient pre-workout supplements (MIPS) [6]. Interpretation of the study on the effects of single active substances is easy and the data can be easier to compare to other reported data in other similar research protocols; in the case of MIPS, this is more difficult due to the different composition of MIPS [6]. Unfortunately, due to the diverse quantitative and qualitative composition of MIPS, studies on the effect of MIPS on physical performance are methodologically much more difficult, and necessarily each type of MIPS requires a separate evaluation of its effectiveness [6]. For this reason, findings to date on the effect of MIPS on performance are often contradictory and inconclusive, in contrast to some pre-workout ingredients as standalone agents, where their efficacy (or lack thereof) in improving performance has been demonstrated quite unequivocally [7]. Consumption of many MIPS is considered relatively safe and minimal side effects are reported following their usage [6]. The enhanced efficacy of MIPS, compared to single substances, is believed to be due to the fact, that certain combinations of ingredients, acting on different physiological mechanisms, can produce a synergistic effect and consequently enhance efficacy and thus significantly improve performance [8].

High intensity efforts i.e. all-out efforts lasting from 6 s to 1 min are performed with predominance of the glycolytic pathway in addition to the phosphagen’s pathway and oxidative phosphorylation [9]. The phosphagen and glycolytic pathways can provide energy very quickly and at very high rates but are limited to short periods of time during high intensity exercise due to substrate depletion and increasing muscle acidosis [10]. Anaerobic capacity is an important parameter for athletic performance, not only for short high-intensity activities but also for breakaway efforts and end spurts during endurance competition [11]. High-intensity exercises are characteristic of many individual or team sports [12]. During anaerobic efforts e.g. sprint, athletes need to generate power output as high as possible in the shortest possible time (peak power), and then maintain it for as long as possible (sustained power), with as little power decrease as possible [13]. In practice, this translates into high speed/strength/power and anaerobic endurance for the athlete [14]. The level of anaerobic perfomance is influenced not only by factors related to the efficiency of the glycolytic and phosphagen pathways (i.e., efficiency of the production of ATP in these two pathways), but also by blood buffer capacity (necessary to buffer lactate) [15], and, due to the short duration of the effort, psychological factors such as focus [16].

MIPS typically contain a blend of ingredients such as caffeine, branched-chain amino acids, nitrates, creatine, β-alanine L-citrulline, vitamins, taurine, guarana and other ingredients in various combinations and proportions [8, 17, 18]. Among the dietary supplements currently in use, there are also those that can increase an athlete’s anaerobic capacity. Caffeine acts as an adenosine receptor antagonist in the brain [19], and has been shown to acutely improve cognition as well as performance during endurance, power, and resistance exercise [20, 21]. β-alanine is a precursor to carnosine, a dipeptide which acts as an intramuscular buffer [22]. Muscle carnosine concentration could be an important factor in high-intensity exercise performance - a significant positive correlation between carnosine concentration and power output was observed in 30 s of maximal sprint cycling [23] and total work done during the cycling capacity test was increased [24]. Taurine plays a beneficial role in diverse metabolic and physiological processes, such as glucose and lipid regulation, energy metabolism, anti-inflammatory modulation, and antioxidant actions [25, 26]. Accordingly, taurine has been used as a potential ergogenic aid to improve athletic performance [26], and previous research has demonstrated taurine may reduce blood lactate accumulation [27]. The reduction in lactate concentration is likely due to a possible interaction between taurine and the role of calcium in mitochondrial buffering - as taurine increases mitochondrial buffering [26]. Taurine also improves aerobic metabolism (it modulates lipid metabolism and stimulates genes responsible for mitochondrial biogenesis), and may improve force and power production via interactions with the muscle membrane and sarcoplasmic reticulum - taurine is essential for excitation-contraction coupling mechanisms [26]. Although another study indicated lower lactate gain in exercise after taurine supplementation, but at the same time, no improvement in performance was observed [28]. Acute tyrosine consumption in moderately trained participants was associated with increased endurance capacity and may have affected the ability to subjectively tolerate prolonged submaximal constant-load exercise in the heat [29]. Capsaicin consumption is leading to the sensation of heat, activation of the sympathetic nervous system [30], with increased catecholamine secretion, fat oxidation, and energy expenditure [31]. Capsaicin supplementation was found to increase time to exhaustion in high-intensity intermittent exercise [32].

Literature and qualitative studies examining the effectiveness of MIPS supplementation are only preliminary [6], and previously published papers reported contradictory data [33–36]. Ingestion of the pre-workout dietary supplement led to significant improvements in anaerobic peak and mean power values measured in Wingate test in recreationally trained males [33], and was effective at increasing both aerobic and anaerobic alactic energy contribution and time to exhaustion during a high-intensity interval exercise in physically active individuals [34]. Another studies [35, 36] found no effect of MIPS on on muscular endurance and Wingate anaerobic capacity sprint performance in recreationally active participants or strength/power athletes. Also, Lane and Byrd [37] showed no effect of caffeine or MIPS ingestion on the cycle sprint performance and vertical jump performance, after both supplements consumption peak velocity in bench press significantly increased. It is presumed that, the synergistic effect of all these substances may have a significant impact on anaerobic performance [8]. We hypothesized, that the ingestion of MIPS containing caffeine, taurine, β-alanine, tyrosine, and capsaicin immediately before exercise may significantly improve anaerobic performance. The aim of our study was to determine the acute effects of commercially available MIPS containing the aforementioned active substances on peak and mean anaerobic power in Wingate test in untrained men.

## Materials and methods

### Study design

A randomized, crossover, single-blind design was used to evaluate the effects of the dietary supplement on peak and mean anaerobic power. The participants performed the supramaximal all-out test (Wingate Test) twice, in random order: as a control test (CTRL) and after MIPS administration (SUP). Blinded participants drew balls of different colors (6 red and 6 blue). The red ball drawn indicated, that the SUP trial would be conducted first, while the blue ball indicated that the control test with placebo would be conducted first. The measurements took place over two consecutive days. The exercise tests took place under medical supervision. A practice effect is observed in Wingate test [38], hence the familiarization session was performed before main maeasurements. In our study, to further minimize this effect, the study was planned as a crossover study. Somatic measurements were taken on the same day that the first all-out test was performed.

The inclusion criteria were as follows: lack of neuromuscular and musculoskeletal disorders; no medication nor dietary supplements used within the previous month which could potentially affect the study outcomes and self-described good health status. Among the inclusion criteria, habitual intake of active substances, e.g. caffeine, should also be considered. Participants’ habituation to caffeine through chronic caffeine exposure may affect the ergogenic effect of acute caffeine consumption [39]. Although the effect of habitual caffeine intake is not clear [40], however, recently published results [41] indicated that, habitual caffeine intake may not have a significant effect on the ergogenic effect of acute caffeine supplementation on resistance exercise, jumping and Wingate performance. Because of these ambiguities, we only recruited participants declaring low and occasional caffeine intake for the study. i.e. only one caffeinated coffee drink per day or less.

All volunteers declaring consumption of energy drinks were also excluded from the study. Prior to the study, participants were advised to maintain standard dietary and hydration habits for all measurement days. Participants were instructed to refrain from all sources of caffeine, alcohol or dietary supplements during the study, not to engage in strenuous exercise for 24 h before the first testing session and during the study, and to recover without additional physical or medical modalities. They should also have slept for at least 6–8 h the night before the test and eat a light meal at least 2 h before the test/training. Participants signed an informed consent form to participate in the project.

The sample size was determined priori using G*Power version 3.1.9.2 (Dusseldorf, Germany), and the following parameters were assumed as a statistical test: t-test, difference between two dependent means (1 group, 2 experimental conditions); the statistical power was 0.8, the significance level was 0.05, and the effect size was 0.8. The power analysis indicated that a minimum sample size of 12 individuals was required for this study.

The project was approved by the Bioethical Commission of the Regional Medical Chamber in Krakow, Poland (opinion No. 90/KB/OIL/2018). All test procedures were conducted in accordance with the principles adopted in the Declaration of Helsinki and the study adhered to CONSORT guidelines.

### Somatic measurements

Somatic measurements were taken on the first day of the study, before the exercise test, in the morning. Height (BH) and body mass (BM) and body composition were measured. Body height was measured to the nearest 1 mm using a stadiometer (Seca 217, Germany). Body mass and body composition were measured using a body composition analyzer (Jawon, IOI-353, Korea) (bioelectrical impedance method). In the body composition measurement, percentage of body fat (%F), lean body mass (LBM) were estimated. Body mass index (BMI) was also calculated for each participant. Body composition was measured with normal hydration of the body, after the feet and hands had been previously degreased (four-limb measurement, 8 measuring electrodes, three measurement frequencies).

### Participants

Twelve young healthy men, aged 22.4 ± 1.44 years, without medical contraindications to supramaximal and maximal intensity exercise, participated in the study. Their height averaged 181 ± 6.4 cm, body mass 77.44 ± 11.8 kg, %F: 16.3 ± 3.2%, LBM: 64.0 ± 8.36 kg and BMI 23.8 ± 3.1 kg/m^2^. Neither of them was involved in competitive sports.

### Supplement

The multi-ingredient supplement Redweiler Shot (Olimp Labs, Debica, Poland) in orange flavor was selected for the study. This supplement is commercially available and dispensed without a prescription. The dietary supplement contained in one dose (30 ml): β-alanine 3000 mg, taurine 1000 mg, caffeine 290 mg, L-tyrosine 125 mg, and cayenne pepper extract (Capsicum annuum L.) 4.2 mg (of which 8% capsaicin). The supplement was mixed with water; in total, participants were given 100 ml of liquid to drink, 30 min before the warm-up for the all-out anaerobic test. As a placebo, participants were given 100 ml of orange-flavored water without any additional substances (0 kcal). Thus, in both conditions, participants were given the same volume of fluid, with the same taste and color, at the same time point before the anaerobic test (single blind).

### All-out supramaximal test – Wingate anaerobic test

The study applied a 20-second version of the Wingate test and test was performed from a stationary start. The test was performed in the morning hours, on a bicycle ergometer (Monark E834, Sweden) equipped with a revolution time meter. Dedicated Wingate test software (MCE, JBA, Poland) was used to calculate the variables investigated. The following variables were measured in the test: total work performed (TW), peak power (PP), mean power (MP), time to reach peak power (TTR-PP), and power decrease (PD) (also referred to as fatigue index). The test was preceded by a five-minute warm-up performed with a load of 90 watts. During the warm-up, the participants performed two maximal accelerations (at the 3rd and 5th minute) lasting about 6–7 s. Following the warm-up was a 5-minute passive recovery. The participants then performed a supramaximal all-out test with a load of 7.5% of body mass. During the test, the participant’s task was to perform a maximal sprint on the ergometer, i.e. to achieve maximum pedaling speed in the shortest possible time and then to maintain that pedaling speed for as long as possible. During the test, the participants were vigorously verbally motivated throughout the test.

A few days before the start of the study, the participants were familiarized with the laboratory, the ergometers and the exercise test procedure. At that time, they also performed a trial effort, similar to the baseline test. The tests took place in the morning, at the same temperature of 21 ± 0.5 °C and humidity of 40 ± 1%.

### Statistical analysis

The distribution of variables was checked with the Shapiro–Wilk test. Data are presented as median (Me), quartile deviation (QD), and lower and upper quartiles (Q1 and Q3). Significance between the conditions was assessed using the non-parametric Wilcoxon tests. Effect size (ES) for Wilcoxon analyses were calculated based on biserial correlation coefficient (r) where r=$Z/\sqrt{N}$ and interpreted according to Cohen’s guidelines for r: large effect is 0.5, a medium effect is 0.3, and a small effect is 0.1 [42, 43]. The differences in the results were considered statistically significant for *p* < 0.05. The STATISTICA 13.1 PL for Windows package (StatSoft, Inc., United States) was implemented for statistical calculations.

## Results

Three participants from SUP group reported paresthesia in the lower extremities, i.e. skin tingling (an effect of beta alanine). The participants did not report any other side effects after consuming the dietary supplement.

### Anaerobic performance

**Fig. 1:**
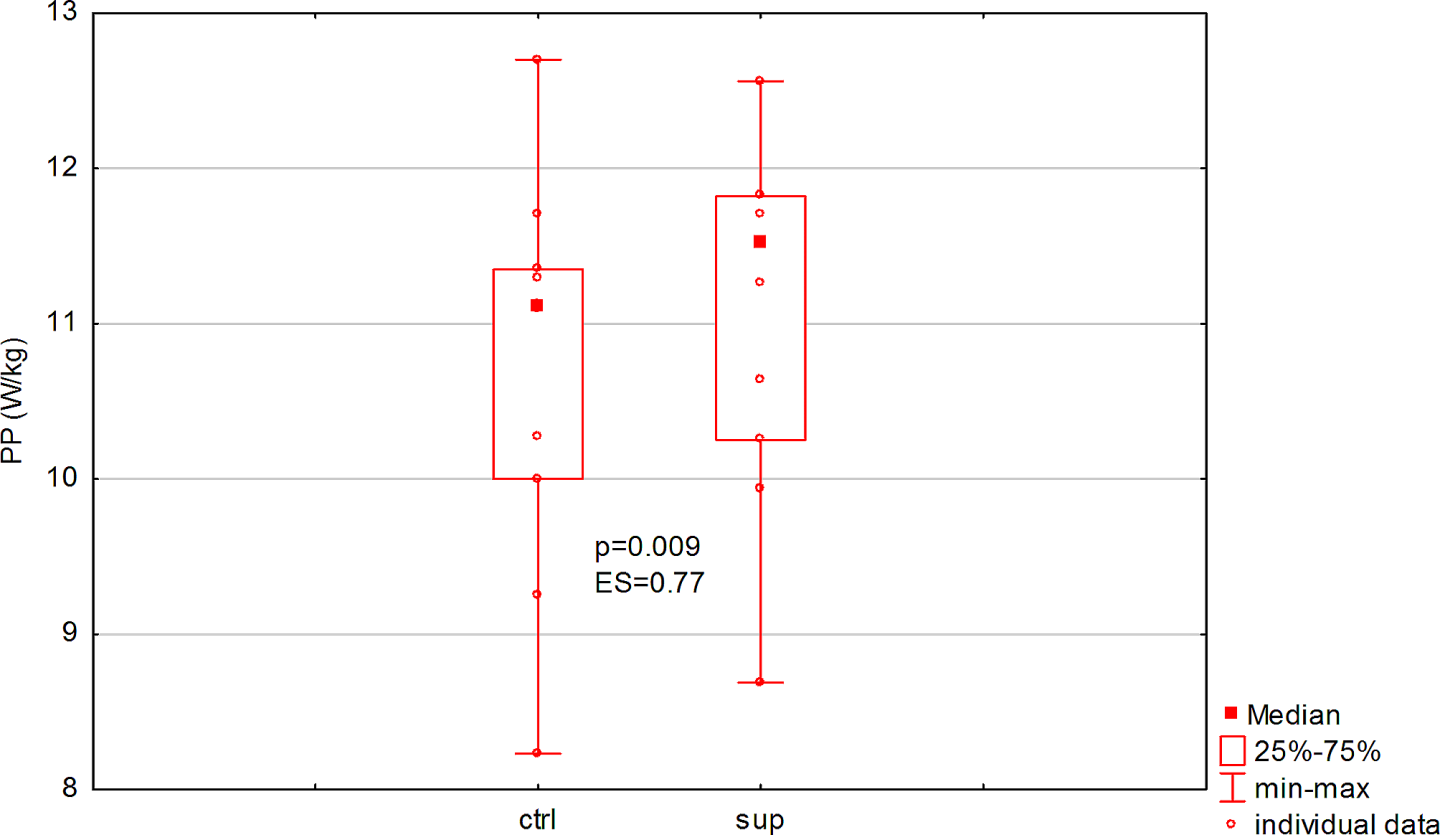
Acute effect of dietary supplement on relative peak power (PP) in men (ES: effect size)

**Fig. 2:**
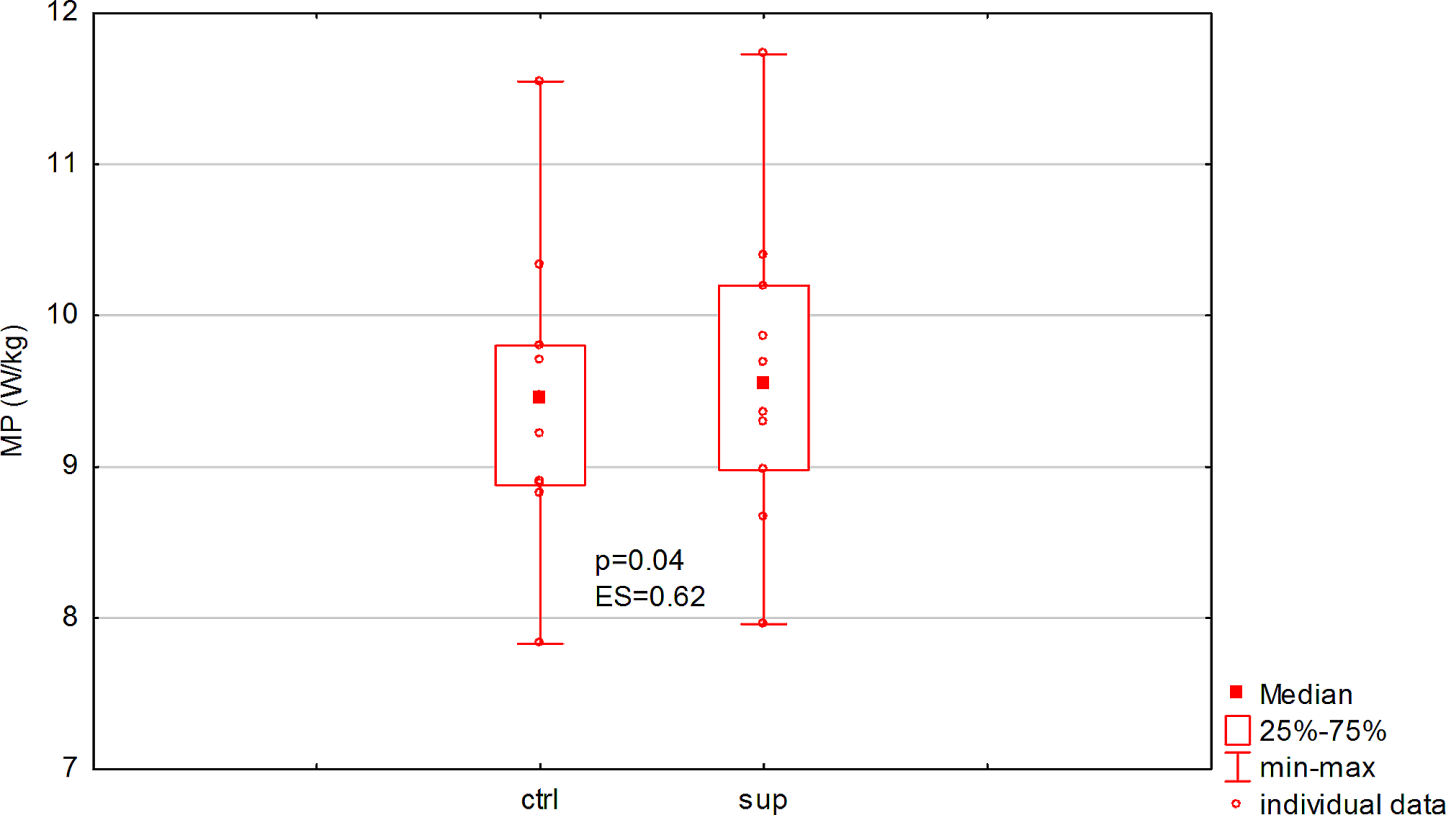
Acute effect of dietary supplement on relative mean power (MP) in men (ES: effect size)

Consumption of the supplement did not significantly (*p* = 0.10, ES = 0.48) affect the amount of total work performed in the test, or the rate of power decrease (fatigue index) (Table 1). On the other hand, there was a significant increase in peak power (*p* = 0.009, ES = 0.77, Fig. 1) and mean power (*p* = 0.04, ES = 0.62, Fig. 2), and a shortening of TTR-PP (*p* = 0.03, ES = 0.88) (Table 1). The observed effect size should be considered as large (Table 1).

**Table 1:** Effects of used dietary supplement on anaerobic performance

Variable	Condition	Me	QD	Q1-Q3	*p*	ES	ES
TW (J)	CTRL	11265.16	556.05	10676.05-11788.14	0.10	0.48	medium
	SUP	11253.13	598.12	10748.19-11944.43			
TW (J/kg)	CTRL	185.98	9.25	177.6-196.1	0.10	0.49	medium
	SUP	187.52	19.9	178.8-198.7			
MP (W)	CTRL	568.73	27.66	533.86-589.18	0.04	0.62	large
	SUP	573.54	36.71	539.88-613.22			
PP (W)	CTRL	668.42	40.55	601.10-682.20	0.009	0.77	large
	SUP	692.47	47.19	616.13–710.50			
PD (%)	CTRL	14.70	3.02	13.11–19.15	0.07	0.53	large
	SUP	14.63	6.6	10.67–17.27			
TTR-PP (s)	CTRL	7.04	1.23	5.55–8.01	0.003	0.88	large
	SUP	5.70	1.18	5.10–7.45			

TW: total work; MP: mean power; PP: peak power; PD: power decrease; TTR-PP: time to reach peak power; CTRL: control (placebo) condition; SUP: supplement condition; ES: effect size

## Discussion

The aim of this study was to determine the acute effects of a multi-ingredient dietary supplement containing beta-alanine, taurine, caffeine, L-tyrosine, and capsaicin (cayenne pepper extract) on anaerobic performance in young untraind men. We hypothesized that a formulation with this composition would be effective in improving peak power, average power and other indices characterizing anaerobic performance. The results of our study, confirmed our hypothesis - we showed a significant improvement in peak power and time to peak power, and average power under supramaximal all-out effort. Supplementation did not significantly affect the rate of power decrease in the test or the amount of total work performed.

In our study, we used a shortened, 20-second version of the Wingate test. In the Wingate test, which lasts 30 s, there is a relatively high proportion of aerobic metabolism of about 18.6% [44]. In terms of total energy provided during a 30 s all-out sprint, ~ 70–80% is provided by anaerobic sources and only ~ 20–30% by aerobic sources [10, 45, 46]. However, during the 25–30 s time period, aerobic metabolism provides ~ 50% of the total energy [46]. The aim of our study was to investigate the effect of MIPS on anaerobic performance, so in order to minimize the contribution of aerobic metabolism to the test, we opted for a shortened version of the Wingate test, lasting 20 s.

Power is the product of strength and speed, so changes in power may depend on the effect of MIPS on strength or speed or both variables simultaneously. In our study, participants’ speed was significantly improved after MIPS administration, as manifested by a reduction in the time to achieve PP. Previous studies have shown that MIPS containing caffeine, although improving power, did not significantly affect strength [33], suggesting that the improvement in power, noted in our study, is the result of improvements in speed rather than strength. The reported improvements in speed could have been influenced by increased motivation and stimulation of the sympathetic nervous system, indicating that it may have been the components of MIPS that were potentially influencing these factors that were crucial [30, 47]. Unfortunately, we did not study these factors in this study, to confirm this assumption further research is needed with the measurement of these variables.

The results reported in our study are difficult to relate to those of other studies that have assessed the effectiveness of MIPS on anaerobic capacity. This difficulty is due to the different MIPS used in previous studies, which differ in quantitative and qualitative composition [6]. This is likely to be the reason for the discrepancies in assessing the effectiveness of MIPS in improving anaerobic performance. Martinez et al. [33] showed significant increases in peak and mean power in Wingate anaerobic test following consumption of MIPS. In a study by Figueiredo et al. [34], acute MIPS consumption was effective at increasing aerobic and anaerobic alactic energy contribution and time to exhaustion. In other studies, no effect of acute MIPS consumption was found on Wingate anaerobic cycling performance [35, 36]. Lane and Byrd [37] also failed to show an ergogenic effect of MIPS or a caffeine-matched placebo on repeated 5 s Wingate cycling performance. The results of studies to date may be inconclusive, not only because of the different MIPS or protocols used, but also because of other factors. In evaluating the effects of caffeine-based dietary supplements on performance, a number of other external factors such as training status should also be taken into account, habitual caffeine use, time of day, age, and sex on caffeine ergogenicity, and genotype [48]. Also, a study with β-alanine administration indicated that external factors such as nutritional intake, sleep patterns, or training status may influence interindividual variability in response to β-alanine supplementation [49].

It is not only the composition of the supplement that is crucial, but an equally important issue that differs between the various MIPS, is the amount of active ingredient in the supplement. The recommended effective relative dose of caffeine is 3–6 mg/kg [40], whereas the usual average caffeine dose used in studies is close to the lower limit of the recommendation at 3.6 mg/kg [50]. In our study, the dose averaged 3.75 mg/kg, which was slightly higher compared to the doses most commonly administered in previous studies [48, 50]. In general, at least about 300 mg of caffeine per dose in MIPS is an acceptable, sufficient dose for most people [50].

Taurine ingestion induced a small to moderate improvement in repeated Wingate performance [51]. At the same time, taurine intake led to greater fatigue during each sprint [51], and yet power output was better maintained between sprints compared to caffeine alone or simultaneous caffeine and taurine intake. The primary mode of taurine action in skeletal muscle appears to be through intracellular membrane stabilisation, increased calcium ions (Ca^2+^) uptake and release and increased sensitivity of the contractile filaments to calcium ions [51]. Taurine-depleted muscle fibres are suggested to fatigue faster than non-depleted muscle fibres due to altered sarcoplasmic reticulum-Ca^2+^ handling. This proposed fatigue mechanisms could explain the higher peak power after taurine ingestion (i.e. greater Ca^2+^ release) and the resultant greater fatigue [51]. In our study, the taurine dose used was 1 g. In contrast, the doses used in other studies are much higher, up to as much as 6 g [52].

The superior efficacy of MIPS, compared to single substances, is attributed to the synergistic effect of the individual components [8]. Meanwhile, some studies have indicated that the synergistic effect of the substances used may be negative [51–53]. A reduction in anaerobic power output was observed with simultaneous consumption of taurine and caffeine compared to caffeine intake alone [51]. However, contrary data was presented by Karayigit et al. [52], who showed that the combined ingestion of caffeine (6 mg/kg) and taurine (1 g) improved both peak power and mean power in female athletes, and at the same time showed that caffeine or taurine used alone, was ineffective in improving Wingate performance. Similar data presented Ozan et al. [53] who indicate that the combined use of caffeine and taurine is more effective in boxers than their single use.

β-alanine supplementation increases muscle carnosine content and, as a consequence, muscle buffering capacity, which allows an increase in high-intensity cycling capacity through a reduction in the impact of H^+^ accumulation on muscle function and fatigue [54]. Carnosine increases calcium sensitivity in muscle fibers, increasing strength and total work done [24, 54]. In our study, we did not observe a significant increase in total work performed in the Wingate test, indicating that β-alanine did not have the expected acute effect on this variable. In the study by Glenn et al. [55], no significant effect of β-alanine supplementation on anaerobic capacity as determined by the Winagte Test was noted, and the only acute dose of β-alanine (1.6 g) reduced rate of perceived exertion during anaerobic exercise. Perhaps this is due to the too low dose of β-alanine used. Typically used mean amount of β-alanine per serving is well below the recommended effective dose [22]. The suggested dose of β-alanine is 4–6 g per day, and it has been shown that consuming it at this dose for 2 weeks improves high-intensity exercise performance. Similar ergogenic effects can be expected, provided MIPS contains sufficient β-alanine [22], however, the acute effects of such doses on anaerobic performance are not known. In our study, this dose was also lower (3 g) than recommended.

Another component found in the MIPS used was tyrosine, which is a precursor for the synthesis of catecholamine and dopamine [56]. Tyrosine supplementation mediates significant improvements in aspects of cognitive function during exposure to stressful environments and the physical/emotional stress nexus [47]. Tyrosine administration in athletic populations has augmented exercise capacity [29] and cognitive function during exposure to exercise-heat stress [57]. The reported data have not been conclusively confirmed [56] ingestion of TYR did notinfluence cognitive function or physical performance during exercise heat stress [36]. Tyrosine supplementation is typically used during prolonged exercise under stressful environmental conditions [56]. To our knowledge, only one study has examined the effect of tyrosine consumption on anaerobic capacity, and it was reported that, acute intake of tyrosine had no significant effect on muscle strength or anaerobic power [58].

One of the components of the MIPS used in this study was capsaicin. Capsaicin supplementation has been found to acutely increase muscular endurance [59], improved lower-body resistance training performance and reduced rate of perceived exertion [60]. It has also been shown that capsaicin may alter motor neuron excitability and motor unit recruitment [61], which may translate into improved sprint-strength-power performance. Moreover, capsaicin (in a dose of 2 × 390 mg) may attenuate neuromuscular fatigue through changes in afferent signaling or neuromuscular relaxation kinetics [62].

The doses of active substances used in the MIPS used in this study were lower than in other such studies or were at the lower end of recommended doses, e.g. the amount of caffeine averaged 3.75 mg/kg (recommended 3–6 mg/kg [50]), the amount of taurine 1 g (used up to 6 g [52]) or the amount of beta alanine (3 g) (4–6 g recommended [22]). Despite these lower doses, the MIPS used proved to be effective in improving Wingate (anaerobic) performance.

Another aspect that, we believe, may affect the proper interpretation of data in studies on the effect of MIPS on performance is the selection and inclusion criteria of study participants and the choice of exercise protocol. In the case of highly trained study participants (athletes), the observed effect of MIPS may be small, as they are already characterized by a high level of performance, often already at their maximal level. Thus, in contrast to untrained participants, in athletes, the additional improvement in performance under the influence of MIPS may be limited, although in the case of caffeine the ergogenic effect is observed in both trained and untrained [21].

The formulation of the MIPS used in this study included substances that induce a variety of physiological effects. In light of the above-described reported data, supplementation with a single substance was not always effective in improving anaerobic performance, and the effects often depended on the dose, protocol used, or selection of study participants. The described single-substance effect could also only indirectly affect peak and mean anaerobic power. Nevertheless, in our study, we noted a significant improvement in anaerobic mean and peak power after MIPS supplementation. However, it is difficult to indicate which substances significantly affected anaerobic capacity or which ones potentially induced a synergistic effect.

### Limitation of the study

The results of our study apply only to non-trained individuals and to MIPS with the given quantitative and qualitative composition. A different composition of MIPS, as well as the doses of the individual components, may induce different effects. We only investigated the effect of MIPS on anaerobic performance as measured by the Wingate test. In other anaerobic tests (jumping tests, running tests), the effects of the MIPS tested may be different. Despite the sample size calculation, there were 12 participants in our study, which may also indicate the need to confirm the results with a larger sample.

## Conclusions

A multi-ingredient pre-workout dietary supplement containing beta-alanine, taurine, caffeine, L-tyrosine, and cayenne pepper (capsaicin) was found to be effective in improving peak power and average power in the Wingate test. However, the supplement did not affect the amount of work performed in the test or anaerobic endurance (decrease in power). The data indicate, that the improvement in anaerobic power after MIPS ingestion was due to a significant improvement in pedaling speed, which was manifested in a significant improvement (i.e. shortening) in time to peak power, compared to the control group.

## Data Availability

All data generated or analysed during this study are included in this published article.

## References

[1] Reyes-Bossio M, Corcuera-Bustamante S, Veliz-Salinas G, Villas Boas Junior M, Delgado-Campusano M, Brocca-Alvarado P (2022). Effects of psychological interventions on high sports performance: a systematic review. Front Psychol.

[2] Malsagova KA, Kopylov AT, Sinitsyna AA, Stepanov AA, Izotov AA, Butkova TV (2021). Sports nutrition: diets, selection factors, recommendations. Nutrients.

[3] Froiland K, Koszewski W, Hingst J, Kopecky L (2004). Nutritional supplement use among college athletes and their sources of information. Int J Sport Nutr Exerc Metab.

[4] Stratton MT, Siedler MR, Harty PS, Rodriguez C, Boykin JR, Green JJ (2022). The influence of caffeinated and non-caffeinated multi-ingredient pre-workout supplements on resistance exercise performance and subjective outcomes. J Int Soc Sports Nutr.

[5] Wardenaar F, Van den Dool R, Ceelen I, Witkamp R, Mensink M (2016). Self-reported use and reasons among the General Population for using sports Nutrition products and Dietary supplements. Sports.

[6] Harty PS, Zabriskie HA, Erickson JL, Molling PE, Kerksick CM, Jagim AR (2018). Multi-ingredient pre-workout supplements, safety implications, and performance outcomes: a brief review. J Int Soc Sports Nutr.

[7] Eudy AE, Gordon LL, Hockaday BC, Lee DA, Lee V, Luu D (2013). Efficacy and safety of ingredients found in preworkout supplements. Am J Health Syst Pharm.

[8] Jagim AR, Jones MT, Wright GA, St. Antoine C, Kovacs A, Oliver JM (2016). The acute effects of multi-ingredient pre-workout ingestion on strength performance, lower body power, and anaerobic capacity. J Int Soc Sports Nutr.

[9] Chamari K, Padulo J (2015). Aerobic’and ‘Anaerobic’ terms used in exercise physiology: a critical terminology reflection. Sports Med – Open.

[10] Spriet LL (2022). Anaerobic metabolism during exercise. Exercise metabolism.

[11] Noordhof DA, Skiba PF, de Koning JJ (2013). Determining anaerobic capacity in sporting activities. Int J Sports Physiol Perform.

[12] Sandford GN, Laursen PB, Buchheit M (2021). Anaerobic speed/power reserve and sport performance: scientific basis, current applications and future directions. Sports Med.

[13] Mero A, Komi PV, Gregor RJ (1992). Biomechanics of sprint running: a review. Sports Med.

[14] Maciejczyk M, Szymura J, Wiecek M, Szygula Z, Kepinska M, Ochalek K (2015). Effects of eccentric exercise on anaerobic power, starting speed and anaerobic endurance. Kinesiology.

[15] de Oliveira LF, Dolan E, Swinton PA, Durkalec-Michalski K, Artioli GG, McNaughton LR (2022). Extracellular buffering supplements to improve exercise capacity and performance: a comprehensive systematic review and meta-analysis. Sports Med.

[16] Li D, Zhang L, Yue X, Memmert D, Zhang Y (2022). Effect of attentional focus on sprint performance: a meta-analysis. Int J Environ Res Public Health.

[17] Cameron M, Camic CL, Doberstein S, Erickson JL, Jagim AR (2018). The acute effects of a multi-ingredient pre-workout supplement on resting energy expenditure and exercise performance in recreationally active females. J Int Soc Sports Nutr.

[18] Kedia AW, Hofheins JE, Habowski SM, Ferrando AA, Gothard MD, Lopez HL (2014). Effects of a pre-workout supplement on lean mass, muscular performance, subjective workout experience and biomarkers of safety. Int J Med Sci.

[19] Fredholm BB, Bättig K, Holmén J, Nehlig A, Zvartau EE (1999). Actions of caffeine in the brain with special reference to factors that contribute to its widespread use. Pharmacol Rev.

[20] Goldstein ER, Ziegenfuss T, Kalman D, Kreider R, Campbell B, Wilborn C (2010). International society of sports nutrition position stand: caffeine and performance. J Int Soc Sports Nutr.

[21] Guest NS, VanDusseldorp TA, Nelson MT, Grgic J, Schoenfeld BJ, Jenkins ND (2021). International society of sports nutrition position stand: caffeine and exercise performance. J Int Soc Sports Nutr.

[22] Trexler ET, Smith-Ryan AE, Stout JR, Hoffman JR, Wilborn CD, Sale C (2015). International society of sports nutrition position stand: Beta-alanine. J Int Soc Sports Nutr.

[23] Suzuki Y, Ito O, Mukai N, Takahashi H, Takamatsu K (2002). High level of skeletal muscle carnosine contributes to the latter half of exercise performance during 30-s maximal cycle ergometer sprinting. Jpn J Physiol.

[24] Hill CA, Harris RC, Kim HJ, Harris BD, Sale C, Boobis LH (2007). Influence of β-alanine supplementation on skeletal muscle carnosine concentrations and high intensity cycling capacity. Amino Acids.

[25] de Carvalho MB, Brandao CFC, Fassini PG, Bianco TM, Batitucci G, Galan BSM (2020). Taurine supplementation increases post-exercise lipid oxidation at moderate intensity in fasted healthy males. Nutrients.

[26] Kurtz JA, VanDusseldorp TA, Doyle JA, Otis JS (2021). Taurine in sports and exercise. J Int Soc Sports Nutr.

[27] Kowsari E, Moosavi ZA, Rahimi A, Faramarzi M, Haghighi MM (2018). The effect of short-term taurine amino acid supplement on neuromuscular fatigue, serum lactate level and choice reaction time after maximal athletic performance. J Res Med Dent Sci.

[28] de Carvalho FG, Barbieri RA, Carvalho MB, Dato CC, Campos EZ, Gobbi RB (2018). Taurine supplementation can increase lipolysis and affect the contribution of energy systems during front crawl maximal effort. Amino Acids.

[29] Tumilty L, Davison G, Beckmann M, Thatcher R (2011). Oral tyrosine supplementation improves exercise capacity in the heat. Eur J Appl Physiol.

[30] Shin KO, Moritani T (2007). Alterations of autonomic nervous activity and energy metabolism by capsaicin ingestion during aerobic exercise in healthy men. J Nutr Sci Vitaminol.

[31] Ludy MJ, Moore GE, Mattes RD (2012). The effects of capsaicin and capsiate on energy balance: critical review and meta-analyses of studies in humans. Chem Senses.

[32] de Freitas MC, Billaut F, Panissa VLG, Rossi FE, Figueiredo C, Caperuto EC (2019). Capsaicin supplementation increases time to exhaustion in high-intensity intermittent exercise without modifying metabolic responses in physically active men. Eur J Appl Physiol.

[33] Martinez N, Campbell B, Franek M, Buchanan L, Colquhoun R (2016). The effect of acute pre-workout supplementation on power and strength performance. J Int Soc Sports Nutr.

[34] Figueiredo C, Lira FS, Rossi FE, Billaut F, Loschi R, Padilha CS (2020). Multi-ingredient pre-workout supplementation changes energy system contribution and improves performance during high-intensity intermittent exercise in physically active individuals: a double-blind and placebo controlled study. J Int Soc Sports Nutr.

[35] Jung PY, Earnest CP, Koozehchian M, Galvan E, Dalton R, Walker D (2017). Effects of acute ingestion of a pre-workout dietary supplement with and without p-synephrine on resting energy expenditure, cognitive function and exercise performance. J Int Soc Sports Nutr.

[36] Hoffman JR, Kang J, Ratamess NA, Hoffman MW, Tranchina CP, Faigenbaum AD (2009). Examination of a pre-exercise, high energy supplement on exercise performance. J Int Soc Sports Nutr.

[37] Lane MT, Byrd MT (2018). Effects of pre-workout supplements on power maintenance in lower body and upper body tasks. J Funct Morphol Kinesiol.

[38] Barfield JP, Sells PD, Rowe DA, Hannigan-Downs K (2002). Practice effect of the Wingate anaerobic test. J Strength Cond Res.

[39] Pickering C, Kiely J (2019). What should we do about habitual caffeine use in athletes?. Sports Med.

[40] Krawczyk R, Krzysztofik M, Kostrzewa M, Komarek Z, Wilk M, Del Coso J (2022). Preliminary Research towards Acute effects of different doses of caffeine on strength–power performance in highly trained Judo athletes. Int J Environ Res Public Health.

[41] Grgic J, Mikulic P (2021). Acute effects of caffeine supplementation on resistance exercise, jumping, and Wingate performance: no influence of habitual caffeine intake. Eur J Sport Sci.

[42] Fritz CO, Morris PE, Richler JJ (2012). Effect size estimates: current use, calculations, and interpretation. J Exp Psychol Gen.

[43] Cohen J. Statistical Power Analysis for the Behavioral Sciences; Routledge Academic: New York, NY, USA, 1988; ISBN 9780203771587.

[44] Beneke R, Pollmann CH, Bleif I, Leithäuser R, Hütler M (2002). How anaerobic is the Wingate Anaerobic Test for humans?. Eur J Appl Physiol.

[45] Jacobs I, Tesch PA, Bar-Or O, Karlsson J, Dotan R (1983). Lactate in human skeletal muscle after 10 and 30 s of supramaximal exercise. J Appl Physiol.

[46] Parolin ML, Chesley A, Matsos MP, Spriet LL, Jones NL, Heigenhauser GJF (1999). Regulation of skeletal muscle glycogen phosphorylase and PDH during maximal intermittent exercise. Am J Physiol Endocrinol Metab.

[47] Deijen JB, Wientjes CJE, Vullinghs HFM, Cloin Pam Langefeld JJ (1999). Tyrosine improves cognitive performance and reduces blood pressure in cadets after one week of a combat training course. Brain Res Bull.

[48] Pickering C, Grgic J. (2019). Caffeine and exercise: what next? Sports Med. 2019; 49: 1007–1030. 10.1007/s40279-019-01101-0. 10.1007/s40279-019-01101-0 PMC6548757 30977054

[49] Esteves GP, Swinton P, Sale C, James RM, Artioli GG, Roschel H (2021). Individual participant data meta-analysis provides no evidence of intervention response variation in individuals supplementing with beta-alanine. Int J Sport Nutr Exe.

[50] Jagim AR, Harty PS, Camic CL (2019). Common ingredient profiles of multi-ingredient pre-workout supplements. Nutrients.

[51] Warnock R, Jeffries O, Patterson S, Waldron M (2017). The effects of caffeine, taurine, or caffeine-taurine coingestion on repeat-sprint cycling performance and physiological responses. Int J Sports Physiol Perform.

[52] Karayigit R, Naderi A, Saunders B, Forbes SC, Coso JD, Berjisian E (2021). Combined but not isolated ingestion of caffeine and taurine improves Wingate Sprint performance in female team-sport athletes habituated to caffeine. Sports.

[53] Ozan M, Buzdagli Y, Eyipinar CD, Baygutalp NK, Yüce N, Oget F (2022). Does single or combined caffeine and Taurine Supplementation Improve Athletic and cognitive performance without affecting fatigue level in Elite boxers? A Double-Blind, placebo-controlled study. Nutrients.

[54] Sale C, Saunders B, Harris RC (2010). Effect of beta-alanine supplementation on muscle carnosine concentrations and exercise performance. Amino Acids.

[55] Glenn JM, Smith K, Moyen NE, Binns A, Gray M (2015). Effects of acute beta-alanine supplementation on anaerobic performance in trained female cyclists. J Nutr Sci Vitaminol.

[56] Coull N, Chrismas B, Watson P, Horsfall R, Taylor L (2016). Tyrosine ingestion and its effects on cognitive and physical performance in the heat. Med Sci Sports Exerc.

[57] Coull NA, Watkins SL, Aldous JW, Warren LK, Chrismas BC, Dascombe B (2015). Effect of tyrosine ingestion on cognitive and physical performance utilising an intermittent soccer performance test (iSPT) in a warm environment. Eur J Appl Physiol.

[58] Sutton EE, Coll MR, Deuster PA (2005). Ingestion of tyrosine: effects on endurance, muscle strength, and anaerobic performance. Int J Sport Nutr Exerc Metab.

[59] Grgic J, Memon AR, Chen S, Ramirez-Campillo R, Barreto G, Haugen ME (2022). Effects of capsaicin and Capsiate on endurance performance: a meta-analysis. Nutrients.

[60] de Freitas MC, Cholewa JM, Freire RV, Carmo BA, Bottan J, Bratfich M (2018). Acute capsaicin supplementation improves resistance training performance in trained men. J Strength Cond Res.

[61] Evans V, Koh RGL, Duarte FCK, Linde L, Amiri M, Kumbhare D (2021). A randomized double blinded placebo controlled study to evaluate motor unit abnormalities after experimentally induced sensitization using capsaicin. Sci Rep.

[62] Giuriato G, Venturelli M, Matias A, Soares EM, Gaetgens J, Frederick KA (2022). Capsaicin and its effect on exercise performance, fatigue and inflammation after exercise. Nutrients.

